# Circulating S100A8/A9 is potentially a biomarker that could reflect the severity of experimental colitis in rats

**DOI:** 10.1016/j.heliyon.2020.e03470

**Published:** 2020-02-29

**Authors:** Kohki Okada, Hiroshi Itoh, Masaki Ikemoto

**Affiliations:** a Department of Medical Technology and Sciences, Faculty of Health Sciences, Kyoto Tachibana University, Kyoto, 607-8175, Japan; b Faculty of Bioscience, Nagahama Institute of Bio-Science and Technology, Shiga, 526-0829, Japan

**Keywords:** Cell biology, Proteins, Biochemistry, Molecular biology, Gastrointestinal system, Physiology, Immunology, Biological sciences, S100A8/A9, Ulcerative colitis, Biomarker, C-reactive protein, Inflammatory cytokines

## Abstract

**Aims:**

The clinical significance of circulating S100A8/A9 (calprotectin) in patients with ulcerative colitis (UC) is poorly understood. We examined whether serum S100A8/A9 is a good biomarker for UC, and whether the serum level is a useful index for the severity of the disease.

**Main methods:**

Experimental animal (rats) were used to verify clinical significance of serum S100A8/A9 as a biomarker. Rats treated with 5% dextran sulfate sodium (DSS) alone (UCR) or with 5%DSS plus tacrolimus (TMR) were subjected to the experiment. The serum concentrations of rat S100A8/A9 (r-S100A8/A9) and other inflammatory biomarkers, such as C-reactive protein (CRP) and inflammatory cytokines, in the both groups were measured using enzyme-linked immunosorbent assays (ELISAs). The tissue damage in the large intestinal tract was visualized by hematoxylin-eosin staining. The relationship between the serum concetrations of these inflammatory biomarkers and the histological scores of the rectal tissue was statistically analyzed.

**Principle findings:**

As determined by the ELISAs, the serum concentration of r-S100A8/A9 in the UCR hardly correlated with those of not only CRP but also some inflammatory cytokines. The deterioration of the rectal tissue, mainly epithelium structure of a large intestine, in the UCR was clearly observed, but was not so severe as that in the TMR. The histological scores of the rectal tissue in the UCR significantly correlated with the serum level of r-S100A8/A9, but not with other inflammatory biomarkers. Furthermore, macrophages actively produced r-S100A8/A9 in response to stimulation with lipopolysaccharide and quickly secreted it in circulation. Therefore, the serum level of r-S100A8/A9 suggestively changes in accordance with the severity of experimental UC.

**Conclusion:**

Circulating r-S100A8/A9 is a useful biomarker for experimental UC, and its serum level correlates with the disease severity as judged by histological score.

## 1 Introduction

Ulcerative colitis (UC) and Crohn's disease are representative inflammatory bowel diseases (IBD) characterized by chronic inflammation and recurrent diseases in a large intestine (Marks and Segal, 2008; Sairenji et al., 2017). Despite tenacious investigation for three decades, the reason why IBD firstly occurs in the rectal tissue of a large colon remains unclear. It has been reported that abnormalities of the genes involved in innate immunity may be one of the causes and be induced by recognizing and/or processing bacterial components, such as NOD2/CARD15, IRGM, and ATG16L1 (Watanabe et al., 2004; Massery and Parkes, 2007; Henckaerts et al., 2008). Abnormality of innate immunity of antigen-presenting cells, such as macrophages, to commensal bacteria in an intestinal tract is important information for diagnosing IBD. The information is probably helpful for the follow-up of patients with IBD (Kucharzik et al., 2006; Lissner et al., 2015). Establishment of an accurate and rapid diagnostics of IBD is strongly desired for preventing further deterioration of IBD; however, various symptoms of IBD have hampered the development of reliable diagnostics so far.

The colonoscopy is currently the best examination to directly observe a large intestinal tract of the patients with IBD; however, it is hard to repeatedly perform this method to the patients because of its unexpected physical and psychological stress. C-reactive protein (CRP) is a representative biomarker for acute inflammatory response, and is frequently used to verify the extent of remission and mucosal healing of IBD (Solem et al., 2005); however, the CRP level does not always correspond to the clinical condition of patients with IBD, which may lead to false diagnosis by physicians.

S100A8, S100A9, and S100A8/A9 (S100 proteins) are recognized as special proteins that are deeply involved in the onset of inflammatory diseases via toll-like receptor 4 or receptor for advanced glycation endproducts (Odink et al., 1987; Manolakis et al., 2011; Meijer et al., 2014; Wache et al., 2015; Okada et al., 2016). We previously reported that rat S100A9 (r-S100A9) was quickly expressed in antigen-presenting cells, macrophages, in the rectal tissues of rats with UC (UCR) induced with 5% dextran sulfate sodium (DSS) (Okada et al., 2015). In contrast, r-S100A8 and r-S100A9 were co-expressed in most macrophages in the rectal tissue of rats in the TMR that were concurrently treated with an immunosuppressive agent (tacrolimus) in the UCR (Okada et al., 2015). This suggests that r-S100A8 negatively regulates excessive immunological function of r-S100A9 in macrophages. Indeed, we confirmed that r-S100A8 significantly suppressed the secretion of interleukin (IL)-6 from macrophages stimulated by lipopolysaccharide (LPS), and that r-S100A8 negatively regulated the onset of UC in transgenic rats (Tg-S100A8) that abundantly expressed r-S100A8 in many organs, such as liver, kidney, intestinal tract, and brain (Okada et al., 2018).

S100A8/A9 (calprotectin) has been reportedly recognized as a sensitive biomarker for inflammatory diseases, such as rheumatoid arthritis, psoriasis, and vasculitis (Benoit et al., 2006; Pepper et al., 2013; Nordal et al., 2016). In addition, the change in its concentration in the stool well correlated with the disease activity in patients with IBD, especially UC (Zamani et al., 2013; Patel et al., 2017). So far, many investigators recognize human S100A8/A9 (h-S100A8/A9) as a useful biomarker for IBD, especially in its active stage (Meuwis et al., 2013; Kalla et al., 2016); however, it was clinically difficult to directly connect the fluctuation of h-S100A8/A9 with the disease activity of UC. In our previous study, we showed the possibility of serum h-S100A8/A9 in as an index of clinical severity of the patients with IBD. Despite such possibility, we couldn't directly show whether the heterodimer reflects histological severity of UC (Okada et al., 2019). Here we developed a preparative enzyme-linked immunosorbent assay (ELISA) for r-S100A8/A9 to measure circulating r-S100A8/A9 in the UCR and TMR, and evaluated clinical utility of r-S100A8/A9, as compared with other inflammatory biomarkers, such as CRP, tumor necrosis factor (TNF)-α, IL-6, and IL-1β. Administration of tacrolimus in model rats with UC seemed to be most suitable in animal experiments because it is widely used as an immunosuppressive agent with low side effects for the body condition in not only the patients with active UC but also experimental rodent models with IBD all over the world (Takizawa et al., 1995; Yoshino et al., 2010; Komaki et al., 2016). In this study, we focused on the relation between the serum level of r-S100A8/A9 and the severity of UC.

## 2 Materials and methods

### 2.1 Ethics statement

Animal experiments complied with the WMA Declaration of Helsinki-Ethical Principles for Medical Research Involving Human Subjects (64th WMA General Assembly; Fortaleza, Brazil, October 2013) and were approved by the ethics committee and animal experiment committee of Tenri Health Care University.

### 2.2 Animals

Wistar rats (WT; male, 9-week-old, 220–250 g/rat) were purchased from Japan SLC, Inc (Shizuoka, Japan). They were kept in captivity for about one week prior to the experiments and were allowed free access to food and water.

### 2.3 Reagents

Two monoclonal antibodies (mAb2H6 and mAb10D11) specific for r-S100A8 and r-S100A9, respectively, were used to establish an ELISA for the heterodimer, in which the two mAbs were provided from Yamasa Corporation (Chiba, Japan). Horseradish Peroxidase (HRP) Labeling Kit-SH and Biotin Labeling Kit-NH2 were purchased from DOJINDO LABORATORIES (Kumamoto, Japan). ELISA kits for inflammatory biomarkers, such as CRP, IL-6, TNF-α, and IL-1β, were purchased from COSMO BIO Co. Ltd. (Tokyo, Japan) to quantitatively determine their serum concentrations in rats. Dextran sulfate sodium salt (DSS, MW: 36000–50000, MP Biochemicals) was purchased from Wako Pure Chemical Industries, Ltd. (Tokyo, Japan). Tacrolimus was provided by Astellas Pharmacology, Inc (Tokyo, Japan). Clinical Thioglycollate Medium (E-MC17) was obtained from EIKEN Chemical Co. Ltd. (Tokyo, Japan). LPS from *Escherichia coli (E. coli)* was purchased from Sigma-Aldrich Co. LLC (Tokyo, Japan). VECTASHIELD Mounting Medium containing 4′,6-Diamidino-2-phenylindole, dihydrochloride (DAPI), streptavidin (STA)-fluorescein 5-isothiocyanate (FITC); and anti-mouse IgG (horse)-Texas Red (TR) conjugates were obtained from Vector Inc. (Burlingame, CA). All other reagents were obtained from Wakenyaku Co. Ltd. (Kyoto, Japan).

### 2.4 Protocols for the animal experiments

**Figure 1:**
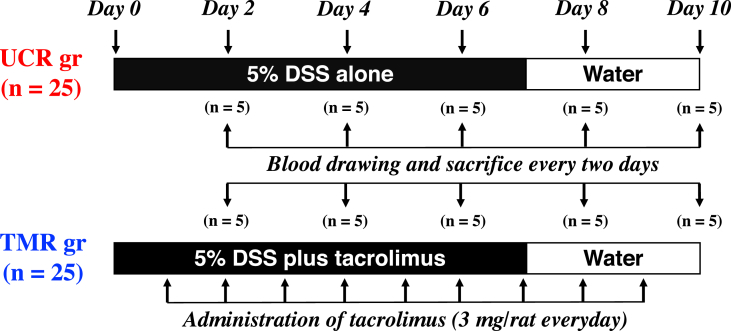
Protocol of animal experiments. Wistar rats (male, 10-week-old, 220–250 g/rat) were treated with 5% DSS alone (UCR) and 5% DSS plus tacrolimus (TMR) during the experimental period, in which tacrolimus (3 mg/rat/day) was orally administrated to the TMR every day as indicated by vertical arrows. Blood samples were drawn from rats in the UCR and TMR. Five rats were sacrificed (n = 5 per day) every two days as indicated by vertical arrows.

Five percent DSS in distilled water (DW) was orally administered to rats (UCR, n = 25) for 7 days to induce experimental UC and then was replaced with DW for the next three days. In the other group, the same protocol as the UCR was employed, in which tacrolimus [3 mg/0.1 mL dimethyl sulfoxide (DMSO)/rat] was also orally administrated to the rats everyday throughout the period (TMR, n = 25). During the period, the body weight of each rat was measured every morning. Five rats in each group were treated as following: blood samples were immediately obtained from the heart under anesthesia every two days. After sacrificing, a large colon of each rat was quickly removed and its length was measured. The large colon was divided into three fragments (rectal, middle, and proximal portion). The segments divided were fixed in 10% formalin/0.1 mol/L phosphate buffer (pH7.4) for histological assessments and then embedded with paraffin. The protocol for the animal experiments is shown in Figure 1.

### 2.5 Enzyme-linked immunosorbent assay (ELISA)

The ELISA for r-S100A8/A9 was performed as described previously (Koike et al., 2012), in which mAb2H6 (5 μg/mL) was used as the primary antibody to capture a subunit (r-S100A8) of r-S100A8/A9, and mAb10D11-HRP (2 μg/mL) conjugate was used as the secondary antibody to capture the other subunit (r-S100A9). The relative amount of r-S100A8/A9 was indicated by the absorbance at 490 nm because it was difficult to prepare stable heterodimers as standards. The concentrations of CRP and inflammatory cytokines (TNF-α, IL-6, and IL-1β) were measured using ELISAs according to the manufacturer's instructions.

### 2.6 Microscopic observation of the rectal tissue of each rat in the UCR and TMR

The thin 3 μm-wide tissue sections that were obtained from the rectal tissues of the UCR and TMR were stained with hematoxylin and eosin (H&E), and the tissue damage was then evaluated histologically (Yoshino et al., 2010). Microscopic images were observed by BIOREVO BZ-9000 (KEYENCE Co. Ltd., Osaka, Japan). Histological scores (HS) were obtained using H&E-staining sections described above. Table 1 shows the criteria of HS based on the previous study (Oh et al., 2017). Rectal tissue close to the anus of rats in the UCR and TMR groups was selected as a common region for HS.

**Table 1:** The criteria of histological scores to a large colon tissue based on HE staining.

Score	Histological findings
0	normal colonic mucosa
1	goblet cell depletion on crypt less than 1/3
2	goblet cell depletion on crypt ranged from 1/3 to 2/3
3	mucosal erosion (partial loss of epithelium with basement membrane left intact)
4	mucosal erosion or ulcer (extensive loss of epithelium including basement membrane) with significant infiltration of inflammatory cells

### 2.7 Isolation of peritoneal macrophages from rats

Peritoneal macrophages were isolated from WT as described previously (Okada et al., 2016). Briefly, macrophages were elicited via the intraperitoneal injection of sterilized 4% thioglycollate/DW (10 mL). After three days, the macrophages were collected in a plastic tube (50 mL) as many as possible using 50 mmol/L sterilized phosphate buffer solution (pH7.4)/0.9% NaCl (buffer A). The tube was then centrifuged for 5 min at 2,329 × g at 4 °C, and the supernatant was discarded. The collected cells in the tube were suspended in 17 mmol/L Tris-HCl (pH 7.2) containing 0.83% NH_4_Cl and incubated for 10 min at 37 °C to make hemolysis any contaminating erythrocytes. After centrifugation as above, the supernatant was discarded, and the residual cells were suspended in RPMI-1640 culture medium containing 10% fetal bovine serum (Biological Industries, Kibbutz Beit-Haemek, Israel; medium B). A total of 2 × 10^6^ cells was plated in each well of a 6-well plate, in which an appropriate volume of medium B had been preliminarily added to each well, and incubated for 2 h in 5% CO_2_ at 37 °C. After incubation, non-adherent cells in each well were removed by washing three times with buffer A. The adherent cells were kept in the same medium in 5% CO_2_ at 37 °C until use.

### 2.8 Activation of macrophages from rats with LPS

After washing macrophages with buffer A thoroughly, the cells were stimulated with LPS (1 μg/mL) for 1 h or 2 h in micro tubes (1.5 mL) at 5% CO_2_ at 37 °C, in which 1 mL of medium B was preliminarily added to each tube, while macrophages treated with equal amount of DW on behalf of LPS were used as negative control. After collecting 1 mL of the medium B into, the cells were used to fluorescent immunochemical staining or collected with 1 mL of buffer A in a polycarbonate tube (15 mL). With regards to 0h stimulation, the culture medium and cells were collected in the same way before washing with buffer A. Then, collected cells were homogenized for 10–15 s at 4 °C and the homogenate was centrifuged for 5 min at 15,880 × g at 4 °C. The supernatant and the culture medium B were subjected to ELISA to measure the intra- or extra-cellular concentrations of r-S100A8/A9 derived from macrophages.

### 2.9 Fluorescent immunochemical staining of r-S100A8 and r-S100A9 in peritoneal macrophages of rats

mAb10D11 was labeled with biotin (long arm)-NH2 using Biotin Labeling Kit-NH2 (DOJINDO LABORATORIES, Kumamoto, Japan). The conjugate, mAb10D11-biotin, was kept in a refrigerator until use. Fluorescent immunochemical staining was carried out as described previously (Okada et al., 2018). Briefly, r-S100A8 was captured by mAb2H6 (5 μg/mL) at 4 °C overnight. After washing three times with buffer A, mAb2H6 binding to r-S100A8 was detected using anti-mouse IgG (horse) IgG-Texas Red (TR). Next, after washing as above, r-S100A9 in macrophages was detected with mAb10D11-biotin conjugate for 2 h at room temperature in the dark. After washing, mAb10D11-biotin complex bound to r-S100A9 was detected with STA-FITC conjugate commercially obtained, which was diluted to 1000-fold by Blocking One/50 mmol/L phosphate buffer (pH7.4), for a half hour at room temperature. After wash three times with buffer A, the macrophages on a cover glass were mounted using VECTASHIELD mounting medium, so that the nuclei of the cells were counterstained with DAPI. Finally, microscopic images were observed using BIOREVO BZ-9000 (KEYENCE Co. Ltd., Osaka, Japan).

### 2.10 Statistical analysis

Pair-wise comparisons with the controls were performed using non-parametric tests. Significant differences between groups were identified using the Mann-Whitney U test. Data are shown as mean ± SD values. Correlation between two groups in each case was assessed by the Spearman test using the statistical software ‘Easy R’ (Kanda, 2013). A correlation coefficient (R-value) between 0.5 and 1.0 indicated good correlation. P-values of <0.05 were considered significant.

## 3 Results

### 3.1 Changes in the body weight and colon length of each rat in the UCR and the TMR after the start of experiment

**Figure 2:**
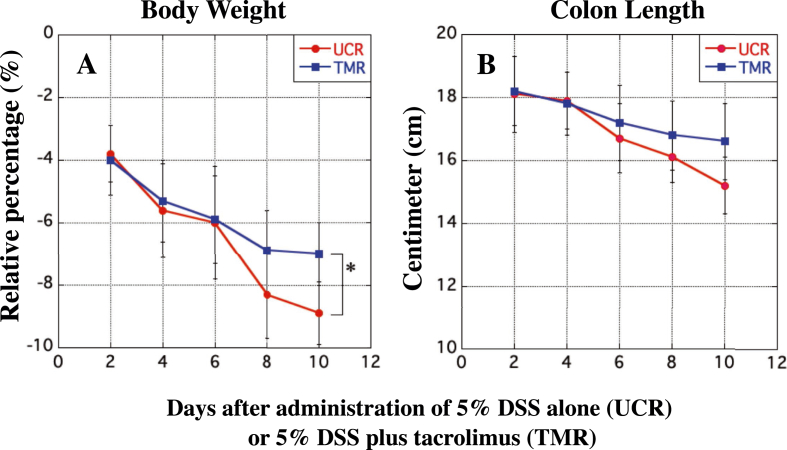
Changes in the body weight and the length of a large colon of rats. The body weight was measured every day. The length of a large colon of the UCR and TMR were measured every two days for the period as indicated by vertical arrows. In A, the y-axis indicates the percentage of decrease in the body weight of the UCR and TMR after the start of experiment. In B, the y-axis shows the length of a large colon of rats in both the two groups after their sacrifice. In A and B, the x-axis indicates the number of days after the start of experiment. Data are the mean ± standard deviation (±SD) (see *Methods*). ∗P < 0.05.

Measurement of the body weight of each rat in the UCR and TMR was carried out every day during the period. Although the body weight in the both groups was gradually decreased over the period, that in the UCR was significantly lower than that in the TMR (Figure 2A). At the end of experiment, the large colon in all rats visually atrophied; however, that in the UCR was slightly shorter than that in the TMR (Figure 2B).

### 3.2 Changes in the serum levels of r-S100A8/A9, CRP, and inflammatory cytokines in the UCR and TMR

**Figure 3:**
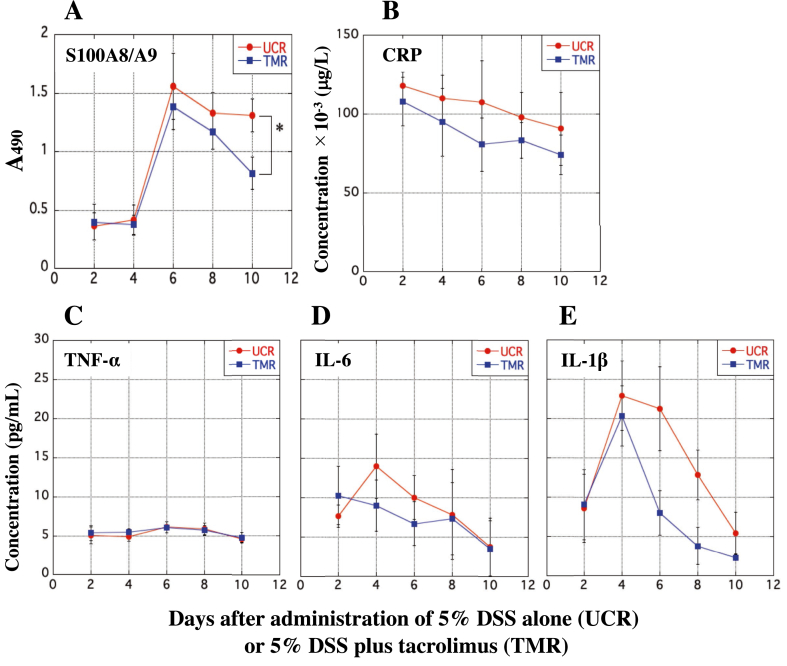
Changes in the serum levels of r-S100A8/A9, CRP, and inflammatory cytokines in the rats treated with 5% DSS alone or 5% DSS plus tacrolimus. The concentrations of r-S100A8/A9, CRP, TNF-α, IL-6, and IL-1β in the serum of the UCR and TMR were measured by ELISAs for each protein according to manufacturer's instructions. In A, the y-axis indicates the absorbance at 490 nm on behalf of the concentration of r-S100A8/A9 in the serum of rats because it was difficult to prepare the heterodimer as a standard. In B, the y-axis indicates the serum concentration of CRP (μg/L) in the UCR and TMR. In C, D, and E, the y-axis indicates the concentrations of TNF-α, IL-6, and IL-1β (pg/mL) in the serum of rats, respectively. In A to E, the x-axis indicates the number of days after the start of experiment. Data are the mean ± standard deviation (±SD) (see *Methods*). ∗P < 0.05.

The serum concentrations of r-S100A8/A9 and inflammatory biomarkers in the UCR and TMR were measured using ELISA. As determined by the ELISA, the serum concentrations of r-S100A8/A9 in the TMR and UCR markedly increased on day 6 after the start and then rapidly decreased; however, its level in the UCR was higher than that in the TMR over the experimental period (Figure 3A). The serum level of CRP in the UCR was also higher than that in the TMR during the period (Figure 3B). In addition, the serum concentrations of TNF-α, IL-6, and IL-1β in the UCR and TMR were measured to evaluate immunosuppressive effect of tacrolimus. In both the two groups, almost no significant increase in TNF-α was found during the period (Figure 3C), but the level of IL-6 markedly increased and reached to the maximum on day 4 and then rapidly decreased (Figure 3D). There was some difference in IL-1β between the UCR and TMR groups on day 4 and day 6, but not significant at all (Figure 3E). Throughout the whole period, the serum levels of IL-6 and IL-1β in the TMR were mostly lower than those in the UCR (Figure 3D and E).

### 3.3 Histological evaluation of the rectal tissue of rats in the UCR and TMR

**Figure 4:**
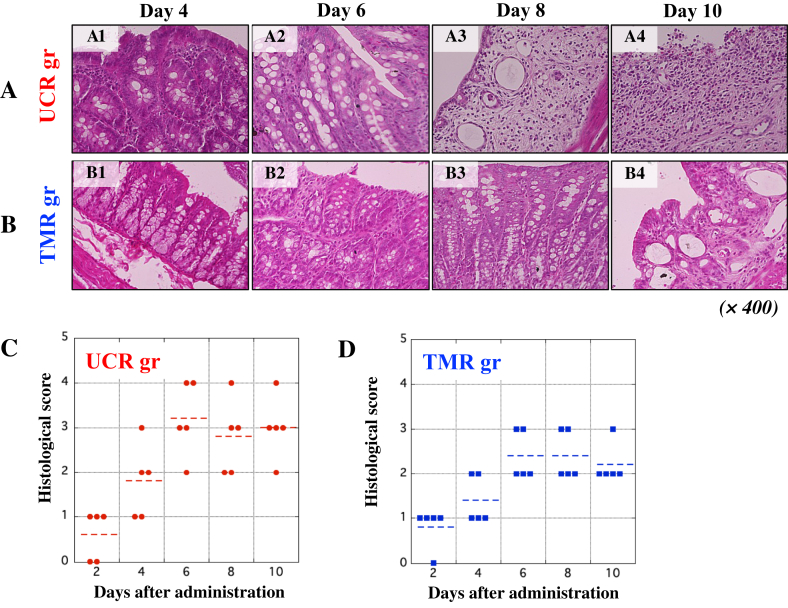
Histological evaluation of large colons of rats in the UCR and TMR by H&E staining. H&E staining was performed to observe the histological changes in the rectal tissues of the UCR and TMR. Panels A and B show the microscopic images of the rectal tissues in the UCR and TMR, respectively. Panels A (A1-A4) and B (B1–B4) indicate the results of H&E staining of the rectal tissues in the UCR and TMR on each day 4, 6, 8, and 10 after the start of experiment. All microscopic images were observed by BIOREVO BZ-9000 (KEYENCE Co. Ltd., Osaka, Japan). Magnification of all panels is high power field (× 400). In C and D, HS was performed using the rectal tissues of the UCR and TMR, respectively, based on HS criteria (Table 1). Data are the mean ± standard deviation (±SD) (see *Methods*).

H&E staining was carried out using thin 3 μm-wide tissue sections of the rectal tissue of the UCR and TMR as described in *Methods*. The extent of tissue damage was histologically evaluated based on the criteria described previously (Yoshino et al., 2010). H&E staining showed that the epithelium in the rectal tissues of the UCR was apparently deteriorated (Figure 4A). On the other hand, the deterioration in the rectal tissues of the TMR was microscopically ameliorated (Figure 4B). In addition, HS in the rectal tissues of the UCR and TMR was evaluated as shown in Table 1 (Oh et al., 2017). After day 4, the value of HS in the tissue of the TMR tended to be lower than that of the UCR (Figure 4C and D).

### 3.4 Correlation between HS and r-S100A8/A9, CRP, or inflammatory cytokines

**Figure 5:**
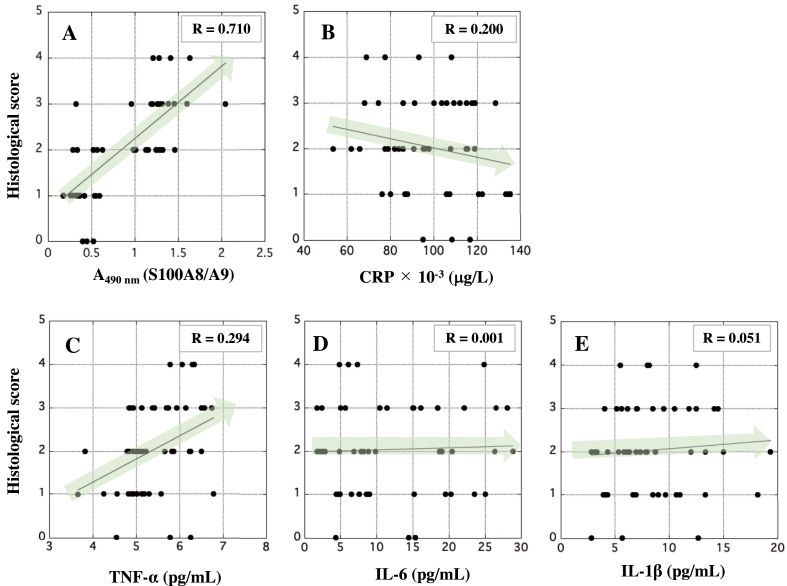
The correlation between histological scores of the rectal tissues of rats and the serum concentrations of r-S100A8/A9, CRP, or inflammatory cytokines in the UCR and TMR. In A, the x-axis indicates the absorbance at 490 nm on behalf of the serum concentration of r-S100A8/A9 because it is very difficult to purify native r-S100A8/A9 or to prepare its recombinant protein with high purity as a standard. In B, the x-axis indicates the serum concentration (μg/L) of CRP. In C, D, and E, the x-axis indicates the serum concentrations of TNF-α, IL-6, and IL-1β (pg/mL), respectively. In A-E, the y-axis indicates the values of HS. The correlations between HS and r-S100A8/A9, CRP, or inflammatory cytokines were assessed by Spearman analysis. R-value between 0.5 and 1.0 is significant statistically.

The correlation between HS and r-S100A8/A9, CRP, or inflammatory cytokines was examined as described in *Methods*. Although the positive correlation between HS and r-S100A8/A9 levels in the serum of the UCR and TMR was seen (Figure 5A), the value of HS negatively correlated with the levels of CRP (Figure 5B). In addition, the value of HS slightly correlated with that of TNF-α, but hardly with those of IL-6 and IL-1β (Figure 5C–E), in which the correlation between the value of HS and the serum level of r-S100A8/A9 in the rats showed the highest R-value (Figure 5A).

### 3.5 Formation of r-S100A8/A9 in macrophages stimulated with LPS and its secretion in circulation

**Figure 6:**
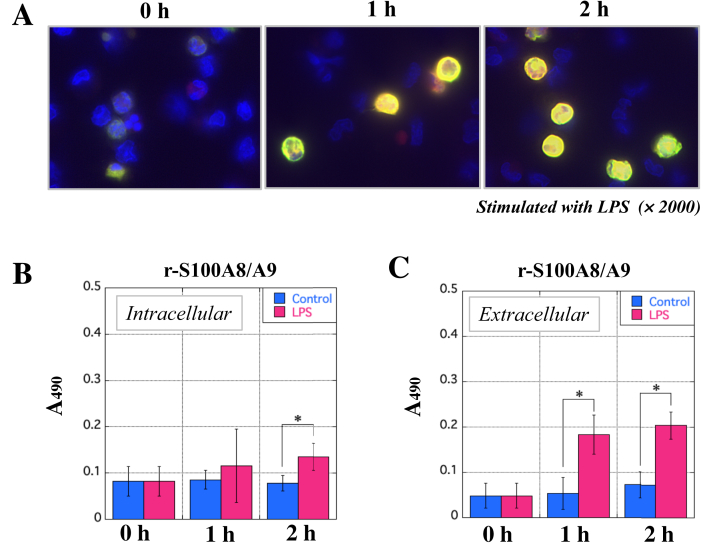
Microscopic observation of intracellular localization of r-S100A8/A9 in macrophages upon stimulation by LPS and the concentration of the heterodimer secreted from the cells. Peritoneal macrophages from WT were stimulated with LPS (1.0 μg/mL) for 1 h and 2 h in 5% CO_2_ at 37 °C. In A, fluorescent immunochemical staining was carried out using the macrophages. The procedures are summarized in *Materials and Methods*. Briefly, r-S100A8 was detected with mAb2H6-biotin and STA-TR, while r-S100A9 with mAb10D11 and anti-mouse IgG (horse) IgG-FITC. r-S100A8 and r-S100A9 were indicated by red and green, respectively, in which the color of yellow indicates microscopic images merged. Nucleus was counter-stained with DAPI (blue). All microscopic images were observed by BIOREVO BZ-9000 (KEYENCE Co. Ltd., Osaka, Japan). Magnification of all panels is high power field (× 2000). In B and C, the x-axis indicates the time points of stimulation with LPS, in which DW on behalf of LPS was used as negative control. The y-axis indicates the absorbance at 490 nm on behalf of the concentration of r-S100A8/A9. Data are the mean ± standard deviation (±SD) (see *Methods*). ∗P < 0.05.

To investigate whether r-S100A8/A9 is formed in activated macrophages followed by its secretion in circulation, we designed an *in vitro* study using peritoneal macrophages obtained from WT. Fluorescent immunochemical staining clearly showed that the expression of r-S100A8 was almost the same as that of r-S100A9 in macrophages stimulated with LPS, but sometimes more dominant in r-S100A9 (Figure 6A). This suggests that r-S100A8/A9 could be formed in the cells. As determined by the ELISA for r-S100A8/A9, the complex in macrophages with LPS was 1.3–1.8 times higher than that in control (Figure 6B). Then, the concentration of r-S100A8/A9 secreted from the cells was approximately 2.8–3.4 times higher than that of control when macrophages were stimulated with LPS (Figure 6C).

## 4 Discussion

In this study, we described a capability of S100A8/A9 as a useful biomarker for UC and a good index for its severity using UC model rats treated with or without tacrolimus and presented data supporting its clinical application. The value of r-S100A8/A9 as a good indicator for IBD is based on its origin and immunological properties. Our purpose is to clear clinical value of circulating S100A8/A9 as a useful biomarker for UC.

We previously reported that the serum concentration of h-S100A8/A9 in patients with IBD was significantly higher than that in healthy volunteers (HVs), and that a positive correlation between the level of circulating h-S100A8/A9 in the patients and the disease activity index (DAI) score was found (Okada et al., 2019). We tried to understand clinical value of circulating S100A8/A9 using rats with experimental UC and found that the serum concentration of r-S100A8/A9 is helpful for histological evaluation of the rectal tissue. Although 5% DSS-induced UC model rats are not always substitute for patients with UC, comprehensive resolution of circulating r-S100A8/A9 is helpful for more accurately understanding its immunological potential using experimental animals. Accordingly, we newly developed an ELISA system for r-S100A8/A9, and applied it to blood samples from the UCR and TMR. As determined by the ELISAs for r-S100A8/A9 and CRP, the changes in the level of r-S100A8/A9 were apparently different from that of CRP in their fluctuation patterns (Figure 3). The serum concentration of CRP in the UCR reached peak value on day 2, while that of serum r-S100A8/A9 reached the maximum on day 6 (Figure 3A and B). Although the difference in the serum concentration of r-S100A8/A9 between the UCR and TMR was only on day 10, the serum level of the heterodimer in the UCR was significantly higher than that in the TMR, indicating that the heterodimer is apparently a sensitive biomarker for experimental colitis. As the extent of UC in the UCR would get worse day by day, the CRP level should increase; however, in spite of the deterioration of UC, the serum concentration of CRP in the rats gradually decreased. The discrepancy suggests that r-S100A8/A9 and CRP do not share in their clinical significance in diagnosis of UC, indicating a possibility that we can explain another advantage of r-S100A8/A9 in clinical application. The mechanism for expression of CRP in animals is not necessarily the same as that in human (Pathak and Agrawal, 2019). Generally, clinical significance of many biomarkers is currently suggested based on the results obtained from animal experiments. As recognized by many investigators, the immunological potentiality of many biomarkers in animals should be suggestive of that of human body.

Next, we investigated the correlation between serum r-S100A8/A9 and other inflammatory biomarkers, such as TNF-α, IL-6, and IL-1β, to clear another potentiality of r-S100A8/A9 as a useful biomarker for IBD, and found that the fluctuation pattern of TNF-α in the TMR was similar to that in the UCR (Figure 3C); however, those of IL-6 and IL-1β were apparently different in the two groups (Figure 3D and E). These results suggest that r-S100A8/A9 directly reflect the severity of UC because the fluctuation of the heterodimer was concomitant with those of IL-6 and IL-1β. Therefore, r-S100A8/A9 may have another advantage over other inflammatory biomarkers for UC.

Histological observation showed severe deterioration of epithelium tissues, accompanied with many immune cells, of the large colons of the UCR (Figure 4A, panels A1 to A4). The epithelium structure of the rectal tissues in the UCR group gradually deteriorated, while normal structure of the epithelium was microscopically maintained in the TMR (Figure 4B, panels B1 to B4). Furthermore, microscopic observation showed that infiltration of immune cells of myeloid origin to the rectal tissues in the TMR was significantly suppressed, and that the onset of experimental UC and its further deterioration were negatively regulated. Thus, tacrolimus is capable of suppressing excessive activation of immune cells to maintain homeostasis in the intestinal tract of rats. The pharmacological performance of tacrolimus has been proved by the fact that tacrolimus negatively regulated excessive activation of lymphocytes and macrophages followed by the decrease in r-S100A8/A9. Concretely, tacrolimus inhibits Ca^2+^-dependent phosphatase (calcineurin) and proliferation of T-cells (Sawada et al., 1987). In addition, tacrolimus leads macrophages to apoptosis by suppressing inflammatory pathways, such as NF-κB, p38, and JNK (Yoshino et al., 2010). Then, activation of macrophages may be suppressed by tacrolimus, so that the immune cells were reduced in a large intestine of rats in the TMR, indicating the therapeutic effects by tacrolimus in the TMR. Indeed, the levels of r-S100A8/A9 complex derived from leukocytes decreased in the TMR (Figure 3A). In addition, the value of HS in the rectal tissues of the TMR was slightly lower than that of the UCR (Figure 4C and D). The therapeutic effect of tacrolimus on suppression of experimental colitis was not so much in our study. The effect of tacrolimus on macrophages excessively activated may be limited in part in animal experiments. Interestingly, the value of HS positively correlated with the concentration of circulating r-S100A8/A9, but negatively with that of CRP (Figure 5A and B). The discrepancy might afford another advantage of r-S100A8/A9 as a sensitive biomarker for UC. The correlation assay between HS and the levels of inflammatory cytokines may be helpful for evaluating the capability of r-S100A8/A9. The value of HS slightly correlated with the concentration of TNF-α, but not with IL-6 or IL-1β as indicated by low R-values (Figure 5C–E). These results indicate that circulating r-S100A8/A9 may be superior to other inflammatory biomarkers to histologically understand intestinal condition of patients with IBD, particularly in active phase. As IBD worsens, a lot of myeloid cells, such as macrophages and neutrophils, should infiltrate in the tissue inflamed. The results of *in vitro* study showed that r-S100A8 and r-S100A9 were actively produced in macrophages stimulated with LPS (Figure 6A). Our data suggests that r-S100A8/A9 may be intracellularly formed in the cells and then extracellularly secreted. To detect the heterodimer, ELISA for r-S100A9 was carried out using mAb2H6-HRP conjugate as the secondary antibody, so that r-S100A8/A9 existed inside and outside macrophages stimulated with LPS (Figure 6B and C). Thus, the formation of r-S100A8/A9 should occur in the cytoplasm of macrophages in a large intestine, in which the heterodimer may be leaked in circulation in patients with IBD. Then, the measurement of h-S100A8/A9 concentration may be useful for correctly evaluating the disease activity in patients with IBD. Furthermore, the measurement of serum S100A8/A9 may be helpful for monitoring the post therapeutic condition of IBD because blood drawing may be easily employed in hospital, compared with the colonoscopy.

The discovery of new biomarkers for IBD in serum, urine, or other specimens would be very beneficial for the patients with IBD. The colonoscopy is clinically effective on early recognition of the disease severity of IBD and its subsequent therapeutic planning; however, this operation put additional strain, such as physical and psychological strains, for the patients, and is time consuming and takes more expensive costs in its operation. In general, urine and stool samples from the patients are widely used in simple laboratory tests to understand the severity of UC because of low cost performance and less invasiveness in the assay (Zamani et al., 2013; Arai et al., 2016; Patel et al., 2017). We examined the localization of r-S100A8 and r-S100A9 in the large intestine of experimental UC rats and their immune functions, and found that the heterodimer was dominantly expressed in macrophages in the rectal tissues of the model rats (Okada et al., 2015). Our data suggests a possibility that r-S100A8/A9, or h-S100A8/A9, could be potentially a biomarker for IBD, particularly UC. The fluctuation of serum S100A8/A9 is observed in various inflammatory diseases, such as rheumatoid arthritis, skin disease and so on. The observation is suggestive of non-specificity for UC (Kang et al., 2014; Benoit et al., 2006). However, many investigators currently insist that S100A8/A9 in stool significantly reflects the severity of UC. In the context, we agree with their concept that the serum level of S100A8/A9 will also change in accordance with the condition of patients with IBD (Zamani et al., 2013; Patel et al., 2017). Then, we focused on the possibility of S100A8/A9 as a valuable biomarker because the heterodimer could be formed from S100A8 and S100A9 in immune cells, or in circulation, and elsewhere in body. The measurement of S100A8/A9 is strongly desired to early diagnose IBD, together with its severity. To overcome the requirements, we have developed an ELISA system for serum r-S100A8/A9 and presented data supporting our idea. The ELISA should allow its easy application to the samples from patients with UC in hospitals.

In this study, we investigated clinical usefulness of circulating r-S100A8/A9 as a biomarker for UC. In conclusion, serum r-S100A8/A9 would be superior to other inflammatory markers as a useful biomarker for UC and as a novel indicator for its histological severity; however, whether the serum level of r-S100A8/A9 directly reflects histological severity in the large colon of rats with DSS-induced experimental colitis is not completely understood. Our effort to scrutinize potentiality of serum r-S100A8/A9 is in progress.

## Declarations

### Author contribution statement

K. Okada: Conceived and designed the experiments; Performed the experiments; Analyzed and interpreted the data; Contributed reagents, materials, analysis tools or data; Wrote the paper.

H. Itoh, M. Ikemoto: Conceived and designed the experiments; Analyzed and interpreted the data; Wrote the paper.

### Funding statement

This work was supported by JSPS KAKENHI for Young Scientists (B: Grant Number JP 17K15786) and Japanese Society of Laboratory Medicine Fund for the Promotion of Scientific Research.

### Competing interest statement

The authors declare no conflict of interest.

### Additional information

No additional information is available for this paper.
